# E. Parmly Brown's Porcelain Bridge-And-Crown-Work

**Published:** 1892-07

**Authors:** 


ARTICLE II.

### E. PARMLY BROWN'S PORCELAIN BRIDGE-AND-CROWN-WORK.

It is not a question as to whether a permanently attached denture to restore lost teeth is the proper thing or not. That has passed. The question now is, what kind of bridge and what kind of crown is the best for the case at hand?

The fact that most dentists are not inserting bridge dentures, is no proof that a large majority would not be practicing the art if they could.

The broad-minded practitioner diagnoses his cases and selects from a large assortment of methods the best treatment of each; the man of one idea always has gold for filling, or, if never gold, amalgam, or always gutta-percha, or always cements.

The same may be said of bridge-and-crown-work.

The bridge-worker who always cuts off his pier teeth is circumscribed in his knowledge and usefulness in the art. Ten cases pass him by unattended to where he operates on one; lacking, as he does, the hardihood to attempt the destruction of good teeth for piers, or failing to get the consent of the patient to attempt such a rash proceeding. The reasons are obvious to bridge-workers; a few cases of denuding fairly good teeth of their enamel, with pulps alive, to make ready for their capping; or amputating such teeth for piers for bridges, satisfy the operator, and he shrinks from any more of that kind of work, which brings more curses than compliments from the patients.

The practice of inserting from one to four or five teeth into gold or amalgam filling attachments will broaden the field of usefulness of the operator.

To say that you have seen failures of fillings holding bridges in place for any great length of time, as an argument against the system, has the same weight as the assertion that you have seen fillings fail, as argument against the wisdom of filling teeth.

I recently extracted a very loose left upper central, from the mouth of a clergyman in New York, in the presence of another dentist, on account of root absorption, which the living central had attached to it and the living cuspid, anchored into gold fillings, a lateral incisor bridge, a porcelain gum plate tooth with soldered gold backing and cross-bar; this bridge had been in its place without repair for eighteen years, having been inserted in 1874 in Salt Lake City, by Dr. Calder.

With modern solid gold and improved gold alloy fillings, and most cases more favorable for good attachment than this presented, who can longer have doubts of the great possibilities of the future in this line.

The fact that your essayist has inserted over a thousand bridges mostly by filling attachment, many of them having been in about eight years, and most of them being under his inspection, accounts for his faith in the practice.

The beginner who, with doubts and misgivings, fails in his attempts, does not prove that one cannot succeed who has become expert by years of study and practice.

Ten years experimenting with porcelain for crowns and bridges has made me a firmer believer than ever in porcelain for most cases; very often using gold crowns for single teeth or roots, or piers for bridges where not in sight, and once in a great while a gold bridge where indicated.

The improved porcelain bridge should rest firmly on the ridge, the surface in contact with which is constructed with a platino-irridium swaged plate, the cross bar and tooth or teeth being first soldered to the plate with pure gold as in continuous gum work; a moderate amount of tooth body first applied, and baked, then full contour obtained at the second baking, gum enamel to finish if necessary, at lower neat, at which baking any small crevices could be filled in with English body, which fuses at about same heat as American gum.

Soft platina caps for ends of roots, either for single crowns or bridge piers (as designed by me), where caps are indicated, made by fitting band, soldered with pure gold, and cut into slits as far as the end of the root, then this aggregation of points is burnished or pressed, one at a time, on to the end of the root, taking its exact form, however irregular; the pin is then pressed to its place, waxed, invested, and soldered with pure gold, unless a porcelain crown is being used with pins, then soldering is not imperative, baking without soldering being sufficient.

The porcelain denture when completed is as cleanly as the natural teeth. It is nearer to nature in form and appearance than any work I know of, and I am satisfied that in the near future, when the facilities for doing the work are to be had, and dentists become conversant with the art, that it will be a delight to patient and operator as well as a profit to both in every way.

The difficulty of the work will tend to increase fees; for that which is easy to do most anybody can do, without much study or effort, and therefore will be done cheaply.

If I could not have porcelain bridges I would be putting in good bridges made on swaged platino-iridium, fitting close to gum or ridge, teeth backed with platina, caps made of platina bars of platino-iridium, square wire, all soldered with pure gold, cap crowns made also of platina and pure gold flowed on them for appearance.

The contour of this structure to be restored as much as practicable to natural form. This would have some of the points of perfection of the porcelain work, lacking mainly an artistic appearance, lacking some in natural contour, some in strength, some in cleanliness, and much in economy of metal and labor. Six points of advantage claimed by the porcelain work over the metal work described, which has the advantage of the ordinary gold bridge that does not rest firmly on the gum, in several respects, principally in the additional support obtained by so resting.

These gold bridges I would insert as I do now the porcelain bridges, mainly with filling attachments, some cemented to root piers, and some to cap crowns.

The question of solid gold fillings to anchor bars, extending from bridges into cavities in pier teeth, is solved by using the Bonwill electric mallet with current from the Edison circuit if possible, if not a strong battery, or the next best force to thoroughly condense the gold.

The tooth to be braced at first by heavy retaining instrument held in left hand till the filling is anchored, then the tooth should be braced by an appliance devised by me, which I have used for several years, made of a bar of tin pointed and curved properly to hold against the tooth malleted on, held either by left hand of operator or by an assistant, which bar is suspended by cord and counterbalance from above, or can be held in hand only.

This metal bar takes nearly all the force used in condensing, and holds the tooth rigid to make the force applied more effective.

The necessity of solid gold fillings to anchor bridges, brings the operator up to a higher standard of well-anchored and solid gold fillings for all his work.—*Review.*

